# A Real-Time Kinect Signature-Based Patient Home Monitoring System

**DOI:** 10.3390/s16111965

**Published:** 2016-11-23

**Authors:** Gaddi Blumrosen, Yael Miron, Nathan Intrator, Meir Plotnik

**Affiliations:** 1 Blavatnik School of Computer Science, Tel Aviv University, Tel Aviv 69978, Israel; nathan@intrators.com; 2 Center of Advanced Technologies in Rehabilitation, Sheba Medical Center, Ramat Gan 52621, Israel; yael_miron@yahoo.com (Y.M.); Meir.PlotnikPeleg@sheba.health.gov.il (M.P.); 3 Sagol School of Neuroscience, Tel Aviv University, Tel Aviv 6997801, Israel; 4 Department of Physiology and Pharmacology, Sackler Faculty of Medicine, Tel Aviv University, Tel Aviv 6997801, Israel

**Keywords:** Kinect, motion tracking, gait analysis, artifact detection

## Abstract

Assessment of body kinematics during performance of daily life activities at home plays a significant role in medical condition monitoring of elderly people and patients with neurological disorders. The affordable and non-wearable Microsoft Kinect (“Kinect”) system has been recently used to estimate human subject kinematic features. However, the Kinect suffers from a limited range and angular coverage, distortion in skeleton joints’ estimations, and erroneous multiplexing of different subjects’ estimations to one. This study addresses these limitations by incorporating a set of features that create a unique “Kinect Signature”. The Kinect Signature enables identification of different subjects in the scene, automatically assign the kinematics feature estimations only to the subject of interest, and provide information about the quality of the Kinect-based estimations. The methods were verified by a set of experiments, which utilize real-time scenarios commonly used to assess motor functions in elderly subjects and in subjects with neurological disorders. The experiment results indicate that the skeleton based Kinect Signature features can be used to identify different subjects in high accuracy. We demonstrate how these capabilities can be used to assign the Kinect estimations to the Subject of Interest, and exclude low quality tracking features. The results of this work can help in establishing reliable kinematic features, which can assist in future to obtain objective scores for medical analysis of patient condition at home while not restricted to perform daily life activities.

## 1. Introduction

Tracking a human subject’s kinematics and characterizing the subject’s behavior at-home plays a significant role in robotics, computer engineering, physical sciences, medicine, natural sciences and industrial academic areas [1,2,3,4,5]. In medicine, pervasive human motion monitoring can improve the rehabilitation process by moving treatment processes from clinics to homes [6], and facilitate the design of treatment plans and follow-up monitoring [7] to reduce the time and cost for patients making round-trips to hospitals. It can improve the accuracy of diagnoses and the overall quality of the treatment of neurological disorders [8]. It can remotely monitor the life activities of the elderly in their homes [9], and in particular it can detect, in real-time, risk situations for the elderly population like falls [10], and can assist the medical staff in hospitals to monitor patients, even at night. Aggregating context information with real-time daily life activities can help provide better services, service suggestions, and changes in system behavior for better healthcare [11].

Human subject kinematic assessment can be obtained by the estimation of the Body Part (BP) positions over time by contact technologies like Inertial Navigation System (INS) [12] or by non-contact systems. This estimation can be used to classify activity level, performance, and activity type. Such analyses can assist in forming an objective score to gauge the severity of symptoms of neurological disorders like Parkinson’s disease (PD) and others [12]. The kinematic patterns of PD subjects can be used to assess PD severity, for instance in a Freezing of Gait (FOG) situation, where effective forward stepping is involuntarily arrested [13]. 

Unlike contact sensors, non-contact sensors are not wearable, do not require battery replacement, and can give information of all the body parts. The most widely used non-contact methods for motion acquisition are videos based on optical technology. Video recording systems can track human subjects [14] and extract various kinematic features [15], track their activity [16,17], and analyze the subject’s behavior [18]. Typically using markers attached to the patient’s body, such systems are accurate and deployed in many gait analysis laboratories [19], but are expensive, limited in range to the laboratory, and are limited to Line of Sight (LoS) conditions and to the markers’ locations. Existing marker-less video systems are less accurate, require multiple synchronized cameras [17], and sometimes still require an expert’s analysis of the resulting video streams [20]. 

Technologies based on electromagnetic [21,22], and ultrasonic technologies [23] can also be deployed for activity monitoring. A narrow band radar has been suggested [24] for the detection and classification of patients’ movements using a gait signature [25]. An Ultra-Wide-Band (UWB) radar was suggested for the acquisition of BPs’ displacement and motion kinematics [26] or to detect human activity in urban environments [27]. A sonar system based on acoustic technology similar to radar was used to produce an acoustic signature, has been successfully used to classify various activity types [23]. However, Sonar and Radar technologies suffer from multi-path fading, are generally limited in range, and cannot provide precise information about the absolute location of different BPs in time and space [28].

The Microsoft Kinect™ (Kinect) [29], is a system that combines optical- and radar-based technologies for human detection, tracking and activity recognition. The Kinect [30] is an active system for the assessment of human activity acquisition that combines an optical video camera and infrared radar technology. It contains a normal Red, Green, and Blue (“RGB”) camera, a depth sensor based on infra-red radar, and a microphone array, simultaneously providing streams of depth signals, RGB images, and audio signals. The Kinect software that processes and aggregates the images from the infra-red radiation line of sight reflections, together with the related color video streams, can reconstruct skeleton-like parts over time [31], and enable capturing human 3-D motion [32]. 

The Kinect’s ability to assess human kinematic data was validated when compared to an optical marker-based 3D motion analysis (e.g., [33]). The accuracy of the Microsoft Kinect sensor for measuring movement in people with Parkinson’s disease was shown to be high compared to the Vicon motion capture system [19]. The Kinect demonstrated varied success measuring spatial characteristics, ranging from excellent for gross movements such as sit-to-stand (Intra-class Correlation Coefficient (ICC) = 0.989) to very poor for fine movement such as hand clasping (ICC = 0.012) [19,34]. The capability of the Kinect to assess reliable spatiotemporal gait variables was shown in [35]. Activity recognition at home with Kinect using depth maps and cuboid similarity feature was described in [36]. Shape and dynamic motion features were derived from Kinect for activity recognition by using temporal continuity constraints of human motion information for each activity and Hidden Markov Model (HMM) [37]. Skeleton-based activity detection and localization using the Kinect enables effective ubiquitous monitoring [38]. In [39], an algorithm to monitor activities of daily living has the capacity to detect abnormal events in the home (e.g., falling). 

Despite its relatively high accuracy rate and its ability to provide full-body kinematic information, the Kinect (versions 1 and 2), still possesses the following deficiencies [5]: (1) Its range of coverage is limited: when a subject comes too near or goes too far from the coverage range, the resulting information is distorted or unavailable [39,40]; and (2) when multiple people cross through the Kinect range, or when one person is closer than or hides the other, the current Kinect application begins an automatic re-detection process. This results in erroneous human identity detection which leads to an inaccurate interpretation of the data [41]. Deployment of multiple Kinect sensors at various locations in the environment can extended the coverage range [42], but requires additional deployment procedure, and their synchronization and the sensor data aggregation, still remains a challenging task [32,42]. Furthermore, the ambiguity of multiple subject estimations, and reference for distortion of skeleton estimation, was not yet addressed.

In the paper in [43], we proposed inspired by radar [26] and sonar signatures [23] to use a Kinect Signature (KS) to differentiate between subjects. The KS is based on features like subject’s size, proportions between different BPs, kinematics profiles, and when possible, subject’s color and voice features. These KS attributes can be assessed in a separate calibration phase, or using a priori knowledge about the Subject of Interest (SoI). This paper extend the work in [43] and proposes set of static features accompanied with computational techniques to be used to overcome some of the existing Kinect drawbacks and form a major step towards using Kinect based continuous kinematic feature assessment at home. The feasibility of the new technology is demonstrated in an experiment setup composed of three sets: (1) Five people were recorded walking in a room, their static features were extracted, and used to validate the capability to distinguish between the different subjects based on their signatures; (2) tracking complex gait patterns of a subject by simulating a PD patient walking with another subject (simulating a medical care assistant or a family member walking with the subject); and (3) hand-tapping on a surface, to demonstrate the capability of the technology to assess kinematic features of the SoI. 

This paper has three contributions: (1) Suggesting a new set of skeleton-based static features that form a unique subject signature; (2) a mechanism to map the different Kinect instances to the SoI (subject of interest); and (3) methodology to detect and exclude noisy skeleton estimations (due to shadowing or getting near the upper coverage limit of the Kinect) and by this to improve the kinematic features quality. Utilization of the suggested system and procedure has the potential to enable reliable kinematic features at-home while doing daily life activities. The kinematic features can be sent in real time to medical care center to monitor patient medical condition objectively and assist in follow-up treatments [44]. This paper is organized as follows: Section 2 describes the methods used in this study. Section 3, describes the results. Section 4 summarizes the results and suggests directions for future research.

## 2. Materials and Methods

**Figure 1:**
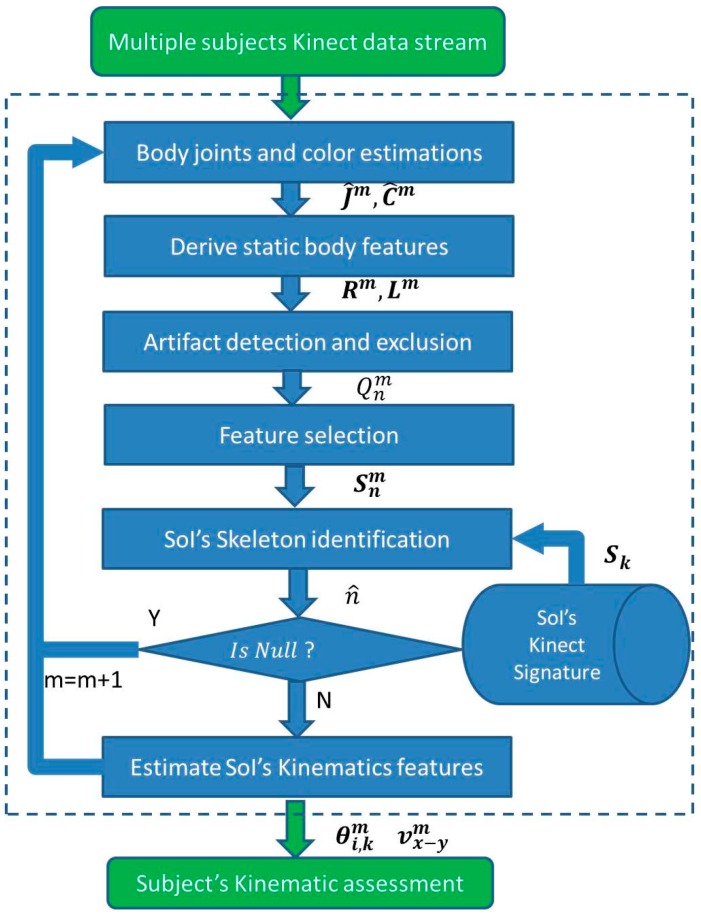
Data analysis scheme flowchart: joint estimation, static features estimation based on the joints’ estimation, feature selection, artifact detection and exclusion in the feature domain, identification of the SoI skeleton based on the KS, and after the KS is assigned to the right subject, kinematic features for kinematic analysis can be derived.

The proposed real-time scheme composed of the following stages: joint estimation, static features estimation based on the joints’ estimation, feature selection, artifact detection and exclusion in the feature domain, identification of the SoI skeleton based on the KS, and ways to derive common kinematic features that can be used for kinematic analysis. All of the data analysis stages are summarized in Figure 1.

### 2.1. Continuous Body Segments Tracking Using Kinect

The Kinect utilizes independent color (RGB) and depth images streams at frame rate of $T_{f}$, which is usually set to 1/30 s [32]. The color and depth images can be aggregated to provide estimations for 3-D joint coordinates (3-D) [45]. The joints coordinates at instance time m, $J^{m}$, can be estimated recursively, similar to [45], by:

(1) $${\hat{J}}^{m}=L\left({\hat{J}}^{m-1},C^{m},D^{m}\right)\;{\hat{J}}^{m}=Kj^{m}+n_{j}^{m}$$

where ${\hat{J}}^{m}$, and ${\hat{J}}^{m-1}$ are the 3-D Kinect joints’ location estimation vectors at time instance *m*, and m−1, $C^{m}$, and $D^{m}$ are the color and depth images at time instance *m*, K, and $n_{j}^{m}$ are the skeleton joints’ distortion, and noise due caused [46], and L is a function that maximizes the joint matching probability based on a very large database of people [5].

Much of the image interpretation is performed independently on each frame, which enhances the system’s ability to recover from tracking errors [45]. Human subjects are identified based on matching their skeleton and depth estimations to a training data set (built from many subjects by Microsoft). The temporarily distortions in the skeleton’s estimations as captured by the term $n_{d}^{m}$, are therefor due to low quality of the measurements, and to discrepancy between the Kinect trained model and its observations, leads to temporal artifacts in the skeleton.

The identified subject becomes part of the current active set, which is restricted to maximum 6 people [5]. The number of joints in the skeleton varies between 20 (Kinect v1), and 25 (Kinect v2) [5]. Still, when one subject hides behind another, or moves in and out of the Kinect range, the skeleton can be distorted. In some cases the skeleton estimation becomes not valid anymore, and a new identification and assignment process is initiated. This can result in erroneous subject’s assignment with an index (Kinect subject instance) that was previously associated with a different subject. This can cause erroneous tracking and lead to degradation in activity diagnosis.

### 2.2. Human Kinect Signature (KS)

For the purposes of this study, the Kinect subject active set is defined as the set of subjects in a given scene. The subject index is assigned blindly by the Kinect to an arbitrary available index. Whenever the subject is excluded from the active set (due to shadowing, or by going outside of the Kinect range) its index becomes available for another subject. Each new subject index assignment forms a new Kinect Instance (KI), which can cause miss-assignment to different KIs.

To consistently assign each identified KI to the right subject in the Kinect database, in particular to the Subject of Interest (SoI), we define a human Kinect Signature (KS) similar to radar [21,22], and sonar signatures [23]. The KS is composed of set of features that their typical sets of values can be used to characterize each subject. These KS characteristics features’ values can be obtained through a calibration or training sessions, with or without deployment of an a priori knowledge about the SoI, and form a KS database of the SoIs [47]. The KS can be used to identify different subjects.

### 2.3. KS’s Feature Set

The KS can be based on static or dynamic features [23]. Static features can be based on: (1) Body dimensions (such as a BPs’ length), body volume, or a BPs’ proportions [48]; (2) consistency of BPs’ colors; (3) facial features; or (4) static features of the voice. Dynamic features are related to gait pattern, stride length, asymmetry in posture, dynamics of the facial expressions, and activity profile. 

The usage of color based features is limited to the time where the subject remains with the same set of clothing, and is affected by change in light conditions. The color of the skin, in areas that are not covered by clothing, like the facial area, can be used alternatively [5]. The use of Kinect facial features, requires advanced and accurate registration, and is not available in many Kinect settings. Skeleton-based features are more reliable, and incorporate the rich database that was used to build Kinect, and include a priori information about human movements. 

The focus of this work is, without loss of generality, on KS based on skeleton-based static features, which has the advantage of having prior knowledge that their value should be consistent values over time. This enables using a low number of features and eases the processing stages. This work focuses on three main skeleton-based static set of features in three domains: BPs’ length, ratio between the BPs’ sizes (both based on estimated joints locations), and body’s color at these joints locations (derived by using the image color related to this set of joints). The first two are based on the assumption of a rigid body [41], and that the relative locations (proportions) and lengths of the subjects’ joints are preserved, and thus should have similar value over time. The color consistency of skin color is high [5], and of clothing is usually restricted to a period of one day. 

As the length of each BP is assumed to be preserved, it can be used to identify each subject [49]. The subject’s BPs spread and body dimensions as derived from depth images, were shown to be informative as body joints features to recognize different human poses [50]. Here we suggest incorporating the sum of BPs’ lengths based on the joints estimations in Equation (1), to provide dimension and length feature. The length’s feature can be defined as:

(2) $$L^{m}=\underset{i,i^{\prime }\in I}{\sum }D\left({\hat{J}}_{i}^{m}-{\hat{J}}_{i^{\prime }}^{m}\right)$$

where the operation D is Euclidean distance metric, I is the full set of joint’s indexes, and $D\left({\hat{J}}_{i}^{m}-{\hat{J}}_{i^{\prime }}^{m}\right)$ is the length of the BP between joints $i^{\prime }$, and i, which is denoted as ${\mathrm{BP}}_{i,i^{'}}$.

Another domain of skeleton features can be based the relative on the joint positions relationship [51]. It can be estimated by the ratio between the BPs lengths. The ratio can be used to differentiate between subjects based on their differences in BPs proportions. The ratio feature at time instance m, can be defined as a subset of ratios between a set of BPs. For subset of two BPs, the ratio between the ${\mathrm{BP}}_{i,i^{'}}$ and the ${\mathrm{BP}}_{l,l^{'}}$, is defined by:

(3) $$R^{m}=\frac{D\left({\hat{J}}_{i}^{m}-{\hat{J}}_{i^{\prime }}^{m}\right)}{D\left({\hat{J}}_{l}^{m}-{\hat{J}}_{l^{\prime }}^{m}\right)}$$

The color features, $C^{m}$, can be obtained by the color coordinates of subset of body areas, based on their related skeleton’s joints [52]. It can use the RGB colors of the joints directly, or it can separate the RGB components by color intensity and the ratio between the RGB colors.

The set of static KS’s features of the *n*’th subject for *M* consecutive samples is given by:

(4) $$S_{n}={\left\{L_{n}^{m},R_{n}^{m},C_{n}^{m}\right\}}_{m=1}^{M}$$

where $L_{n}^{m},R_{n}^{m}$, are the length, and ratio features computed for the *n*’th KI in Equations (2) and (3).

Kinect-based skeleton estimations suffers from three main distortion sources: (1) When the subject moves out of the Kinect effective range and the Kinect uses inaccurate interpolation; (2) erroneous skeleton merges with nearby subjects or objects; (3) inaccurate interpolation, where the subject is partially hidden by an object in the room.

The temporal distortion in the skeleton estimations in Equation (1) leads to temporal artifacts in reference to the KS’s features as derived from any non-noisy skeleton measurements. Since the KS’s features are based on static features that without distortion invariant to different body postures and positions, the KS’s features can be modeled as signal distributed around the distortion free features’ value, which can be seen as the features’ reference value. The KS’s feature set related to the *n*’th KI, at instance time m can be modeled as:

(5) $$S_{n}^{m}=S_{n}^{r}+n_{s}^{m}$$

where $S_{n}^{r}$ is the reference KS calculated based on reference value of the skeleton, and $n_{s}^{m}$ is noise vector at the size of the feature set, and can be assumed as colored noise, due to the correlation between consecutive Kinect estimations.

### 2.4. KS’s Feature Pre-Processing: Artifact Detection and Exclusion

An artifact detection algorithm is applied on the KS’s features to estimate and exclude the temporal distortion factor of the KS’s features, $n_{s}^{m}$, by using the reference KS’s set of values. The reference KS’s features estimation, $S_{n}^{r}$, can be estimated by using another sensor modality like video, where the markers are set at similar point of the Kinect skeleton. The work in [19] has shown that the Kinect joint’s estimations can be accurate with an error in a range of few millimeters under the conditions of: (1) Optimal range from the Kinect (2–3 m); (2) his/her all body is included in the Kinect frame; (3) and in subject in frontal orientation to the Kinect, was shown. Under the assumption that the distortion tracking interval is long enough and its distribution is ergodic, the reference KS’ features can be estimated by the median of the KS’s features value along the life span of the KI. Thus the distortion factor of the *n*’th KI, at the *m*’th time instance, can be estimated by:

(6) $${\hat{n}}_{s}^{m}=S_{n}^{m}-{\hat{S}}_{n}^{r}$$

where ${\hat{S}}_{n}^{r}$ is the median vector of the KS’s features along the *n*’th KI life span in Equation (4). 

In this work, without loss of generality, we use a binary quality measure, to distinguish between low quality measurements that should be excluded from the feature set that is used for identification. A binary quality measure at the *n*’th KS at instance time m, can by two-values confidence level of the estimations defined as:

(7) $$Q_{n}^{m}=\left|\begin{array}{cc} 1 & \|{\hat{n}}_{s}^{m}\|<\varepsilon_{A}\\ 0 & else \end{array}\right|$$

where $\varepsilon_{A}$ is the distortion threshold, which is a function of the KS’s joint estimation error, $n_{j}^{m},$ standard deviation. 

The distortion threshold value should be tuned to maximize the artifact detection probability, and has typical values that move between 0.5 and 3 standard deviations, according to the environment [43]. Tuning the distortion threshold, $\varepsilon_{A}$, should be done in a way to minimize the identification error in the desired environment. Its value increases with deviations from the optimal Kinect range, with the rate of shadowing and rapid movements, where more joints are interpolated, since interpolated joints are likely to be more distorted, and are therefore less reliable. 

More enhanced algorithms can aggregate to the quality measurement higher statistical moments, or abnormal skewness or Kurtosis values [53]. In addition, the artifact detection algorithm can incorporate knowledge about invalid postures, using a priori constraints of the human body [54], about the operation range (if it resides in the optimal coverage), and about the features that are derived by interpolation, mainly when parts of the human body are out of the Kinect frame. The low quality erroneous features that are suspected as artifact are excluded from the KS’s feature set, prior to the subject identification. The low quality measures can be replaced by the KI’s median value ${\hat{S}}_{n}^{r}$, which is the operation of median filtering. Another way to mitigate over the artefactual features, is by using physiological constrains on the skeleton as in [55]. 

### 2.5. Features Selection

After features cleaning, a feature selection algorithm that chooses the more robust features for subject identification and that are less sensitive for the joints’ estimation error, can be applied. Unsupervised feature selection algorithm like sparse Principal Component Analysis (PCA) that choose significant features in the PCs representation based on the features’ variability [56], can be chosen when there is not training data. Alternatively, supervised feature selection like AdaBoost that minimize the training error by using a set of weak classifiers of partial sets of the features [57], or Fisher criterion, which is optimum for Fisher’s linear discriminant classifier, and has computational and statistical scalability [58] can be used. 

### 2.6. Identification of Soi’s Skeleton

The identification of the SoI’s skeleton out of the KIs can be seen as part of classification problem of the KIs’ features to the different subjects. The classification uses the KS’s features of the different KIs. It can be further improved by taking into account the temporal quality of the measurements in Equation (7). 

The classification problem can be reduced to detection of only the SoI’s KIs out of the all KIs in the active set. Without loss of generality, we would choose one (the *k*’th) subject SoI out of the total number of subjects in the Kinect subject active set list. A detection criterion based on minimal square root error criterion for choosing the KS of the SoI from the N-size active set KSs at instance time *m* is:

(8) $$\hat{n}=argmin_{n}\|S_{k},S_{n}^{m}\|\;s.t.\;\|S_{n}^{m}-S_{k}\|<\varepsilon_{D}$$

where the ‖ ‖ operator is the Euclidean distance metric, $S_{k}$ is the SoI’s KS, which can be estimated by reference sensor or by the median of the features’ value in calibration process in optimal Kinect conditions, $S_{n}^{m}$ is the *n*’th KI’s set of features in the active set, n=1, ..., N after artifact exclusion, and $\varepsilon_{D}$ is the detection threshold of subjects in the data base.

The detection threshold can be tuned to minimize the error of subjects of interest in the data base, sometimes referred to as subjects in a white list. In case that artifact was not filtered out, or the subject does not exist in the data set, the estimated index, $\hat{n}$, would be null. 

### 2.7. Kinmetic Features

To enable patient kinematics’ analysis at home, after the SoI’s identification, its related Kinect estimation can be aggregated to one data base, and we can derive skeleton based features for motion and activity analysis. For the purpose of demonstration of the kinematics’ analysis based on Kinect features at home like environment, we will use the common features of joints’ angular orientation [59] and velocity [60]. Other more enhanced features for kinematic analysis, like pose changes over time [61], asymmetry measures, and kinetic pattern can be used to enhance kinematic analysis, and be used to enhance the subject identification by forming kinematic signature [62].

The angular position is based on the angles between two BPs and a common joint (vertex). It can be relative angle, such as the intersecting lines of two BPs and their common joint, or absolute, relative to a reference system. The relative angle between the body parts ${\mathrm{BP}}_{i,i^{'}}$, and ${\mathrm{BP}}_{k,i^{'}}$, in degree, is defined as:

(9) $$\theta_{i-i^{\prime },i^{\prime }-k}^{m}=\tan^{-1}\left(\|\frac{\left({\hat{J}}_{i}^{m}-{\hat{J}}_{i^{\prime }}^{m}\right)\times \left({\hat{J}}_{k}^{m}-{\hat{J}}_{i^{\prime }}^{m}\right)}{\left({\hat{J}}_{i}^{m}-{\hat{J}}_{i^{\prime }}^{m}\right)\cdot \left({\hat{J}}_{k}^{m}-{\hat{J}}_{i^{\prime }}^{m}\right)}\|\right)$$

where the operators ×, and · are the cross and dot products, and ‖ ‖ is the Euclidean norm.

The subject absolute velocity can be estimated by the torso in *x*-*y* plane (usually ground plane) by:

(10) $$v_{x-y}^{m}=\frac{1}{T_{f}}\left(\|{\hat{J}}_{\mathrm{Torso}\left(x,y\right)}^{m}-{\hat{J}}_{\mathrm{Torso}\left(x,y\right)}^{m-1}\|\right)$$

where ${\hat{J}}_{\mathrm{Torso}\left(x,y\right)}^{m}$ is the subject torso x, and y coordinates at time instance *m*.

Following the stages above ensures that the kinematic features are of the SoI. The quality measure of time instances with lower reliability could be aggregated to the kinematic analysis algorithm. Attentively, artefactual time instances of the features with low estimation quality can be mitigated by using interpolation using spatial-temporal correlations. Alternatively, the mitigation of artefactual time instances, can be performed in the joints domain, using an algorithm that inforce physiological constrains on the skeleton as in [61], or by using statistical information on the skeleton data [62], and then to recalculate the features. The “clean” kinematic data can be then used for more accurate activity recognition [63] by applying methods like dictionary learning of the set of the activities [26].

### 2.8. Implementation Scheme

The implementation is separated to two phases: a training phase to assess the SoI’s KS; and a real-time SoI tracking phase, with estimation of kinematic features that can be used for the kinematic analysis. A more general implementation would continuously update the Kinect signature from the tracking data, and additional kinematic features. When multiple Kinect sensors are available in the environment, the same implementation scheme can be applied on each sensor separately, and then combined by choosing the Kinect sensor with the best subject has maximal observance.

### 2.9. Experiment Setup

The motion sensor was a Microsoft Kinect (Kinect v1), which consists of a depth sensor based on an infrared (IR) projector and an IR camera, and a color camera [32]. Since all versions of Kinect (Kinect v1, v2) lack skeleton identification and suffer from skeleton distortion, the same methods can be applied to all Kinect models, and to other skeleton-based sensing modalities. The sensor has a practical range limit of 0.8−3.5 m distance. The angular field of view was 57° horizontally and 43° vertically. The sensor could be tilted up to 27° either up or down. The analysis software was Matlab (version 2015.a, Matlab Inc., Natick, MA, USA), with corresponding Kinect SDK version 1.6 (Microsoft, Redmond, WA, USA) to collect the Kinect estimation. The RGB frame rate, Fs, was of 30 Hz, and the image resolution was of 640 × 480 pixels. For configuration, the trigger time for calibration was setup to 5 s, and for tracking 3 s. 

For evaluation of the quality of the KS features, an offline calibration phase, performed once before the start of the tracking, (similar to taking an identification (ID) photo), is performed. It is sufficient to estimate only the SoI’s KS, as other subjects’ data can be excluded from the analysis based on their distance from the SoI’s KS in Equation (8). In this phase, the SoI is standing in front of the Kinect, in optimal range from the camera (around 3 m [32]), where the full skeleton is included in the image frame in line of sight conditions, and the skeleton distortion is minimal. The features are smoothed, and their mean and standard deviation are calculated. The KS can be estimated by the median of the KS’s features. Feature selection is used to choose the best ratio, and spread features. This stage results in SoI’s KS, and in confusion matrices, that indicates on the success rate of the identification. The SoI’s KS are then stored in database for tracking stage [63].

In the tracking phase, a double buffer mechanism is implemented, wherein the time between each data collection is used for calculating and storing the static features in a buffer for all the skeletons in the active set. Only the skeleton data is stored to minimize the memory resources. In cases where the activity’s context is necessary for the analysis, the stream of video frames can be used after the desired compression. The artifacts are identified and removed, and the estimations are classified to reliable or unreliable using Equation (7). Then the KI of the SoI is identified using the criterion in Equation (8). The identification and artifact removal can be performed using k-means clustering with a delay equal to the number of frames in the block [64], and the data can be sent in real time to central analysis center for continuous assessment of the medical conditions [44]. 

The human tracking mode was set to postures of standing/walking position. The feasibility of the new technology was demonstrated with three experiment sets in two locations. The first experiment set was at a single room (at Tel Aviv University, Tel Aviv, Israel), where five adults subjects (three adult males, and two adult females, the fifth subject, is the author (GB)), were recorded by the Kinect separately. The second, and third experiment sets, were recorded in a room (at the Neurology Department, Tel-Hashomer Hospital, Ramat Gan, Israel) and included two of the authors (GB, YM). The environment simulated a home environment, with challenging the Kinect with different coverage ranges, coming in and out of the camera range, and shadowing. 

### 2.10. Experiment Sets

The feasibility of the new technology and the methods validation, were demonstrated in an experiment setup with three sets that represent daily activities commonly used to examine PD patients’ condition. The first experiment set included five subjects walking randomly in a room and was designed to produce statistics for the subject identification algorithm, and verify the significance of the chosen features. In this experiment, the subjects’ static features were extracted, and used to validate the capability to distinguish between the different subjects based on their signatures; the second experiment set included two subjects that maneuvered around a hall and performed different activities. It was designed to demonstrate the capabilities of the suggested methods to: (1) Detect the set of features that are used for classification; (2) identify and re-identify the SoI based on SoI’s KS from the calibration phase; (3) reject different artifacts (going in and out of the Kinect range and shadowing); (4) give indication regard the tracking quality and reliability based on skeleton distortion; and (5) validate Freezing of Gait (FOG) detection during the turnaround.

**Figure 2:**
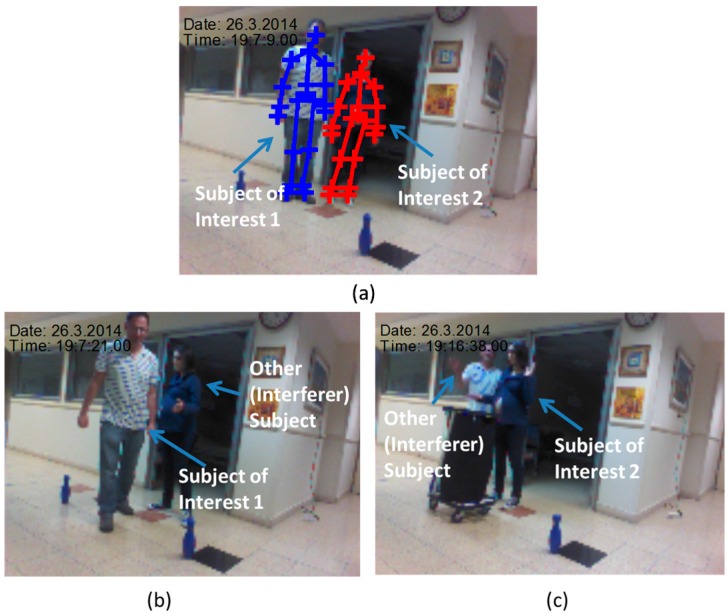
Second and third experiments’ sets: (**a**) Calibration; (**b**) tracking; and (**c**) tapping tracking. In the calibration phase, the two subjects were identified by the Kinect, their features were calculated, averaged, and stored, and formed the KS. The SoI (subject of interest) for the first experiment was the left subject, and for the second, the right subject. For the tracking, the two subjects moved along and out of the range of the Kinect.

At calibration phase the two subjects stood for 7 s. Their KS was derived and stored for identifying the SoI at the tracking phase. Figure 2 describes the calibration and the tracking phase from snapshots of the Kinect video camera. The subjects were walking randomly in the room for around 30 s with the following activities: (1) Walking in a complex pattern in relatively limited space; and (2) repetitive hand tapping. These activities are similar to experimental paradigms which have been recently employed to test motor function of subjects with PD [34]. One subject simulated a patient (the SoI), and the other simulated an accompanying person (an interferer subject), like a medical staff (gait labs), or a non-expert family member (at home). In the tapping experiment the SoI was the second subject (Figure 2a), and the other subject simulated an interferer subject in the scene. The path was marked by bowling pins (Figure 2b). The third experiment set was composed from hand tapping activity. 

Tapping is widely used as a measure of bradykinesia in Parkinson’s disease (PD). In addition, parkinsonian subjects, in general, tap more slowly than non-PD patients, more slowly in the more affected arm, and do not benefit as much from continued practice as do normal subjects, due to impairment of procedural motor learning in PD [64]. The hand-tapping on a surface as shown in Figure 2c, was designed to demonstrate the capability of the technology to assess SoI’s kinematic features that can be used for commonly used kinematic analysis. The kinematic features accuracy, in particular the ones of spatiotemporal gait variables [35], or of the limbs, of time instances where the Kinect estimation are of high quality, can be assumed to be high and be used to assist in diagnosis of PD condition [19].

## 3. Results and Discussion

### 3.1. Choosing the KS’s Features, and Evaluating Their Accuracy

The static features of different BP lengths and ratios, using 34 permutations of the joints as shown in Table A1, were calculated according to Equations (2) and (3). The artifacts were removed using a median filter with $\varepsilon_{A}=1.4$. A Discrete Function Analysis classifier was used with *K*-fold 10, to quantify the separation between the different subjects. The two highest features of ratio, and of length, based on PCA feature selection with dimensionality knowledge enforcement [56]. The length features was the sum of the four limbs, which is correlated with the central body area in [49], and the ratio was of the one between the distance of the two shoulders and the spine length. 

**Figure 3:**
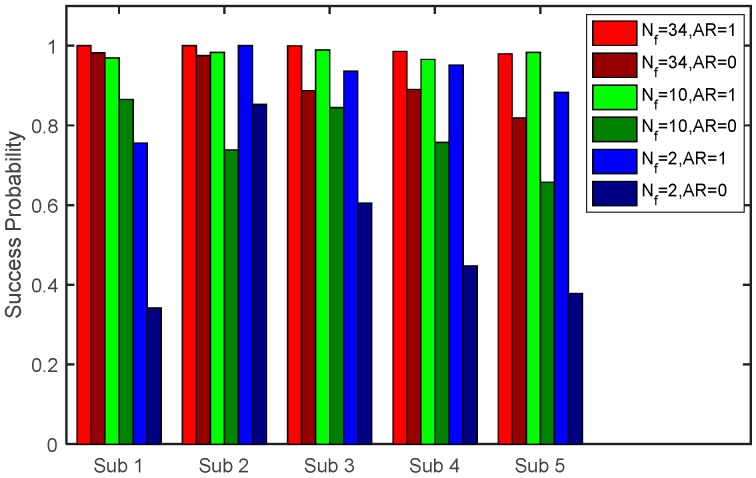
The effect of features selection and artifact removal on the subject-identification success probability. The accuracy declines with the number of features (*N*_f_) and increases when artifact removal is applied (AR = 1). With artifact removal, with only two features, a feasible classification success rate of rate of over 90% can be achieved.

The classification success probabilities for all 34 features (*N*_f_ = 34, 21 length, 13 ratio), 10 features (five length, five ratio, randomly chosen), and for the two features (sum of the four limbs, and ratio between the shoulders and the spine length), with and without the artifact removal, are summarized in Figure 3. Without artifact removal, the classification success rate with all features was of 0.915, which is considerably high, 0.773 for the 10 features, and for the two chosen features only 0.525. The success rate of 0.525, is still much higher than the random selection (0.2), but can be not adequate for some applications. Applying the artifact removal algorithm in Equation (7), with $\varepsilon_{A}$ = 1.4, with all features, has raised the classification success rate to 100% (average of 0.993). The success rate decreased with using 10 features by around 2% (0.978) and with two skeleton features by 9% (0.905). Table B1, Table B2 and Table B3 shows the confusion matrices. The rows describe the true values, and the columns the estimations. It is shown that with all features, almost all subjects’ frames are identified correctly. The identification success decreases with the decline in feature number, not uniformly, for example the first and fourth subject.

The classification results indicate that: (1) The subject identification becomes more accurate with higher number of features; (2) that the artifact removal is significant for more accurate KS, in particular with lower number of features. Adding more features, like facial, dynamic, or color features, if available, is expected to further improve the classification accuracy. Still, for better visual interpretation and to demonstrate the use of the suggested method, only the two skeleton features are selected.

### 3.2. Establishing the SoI’s KS through Calibration

**Figure 4:**
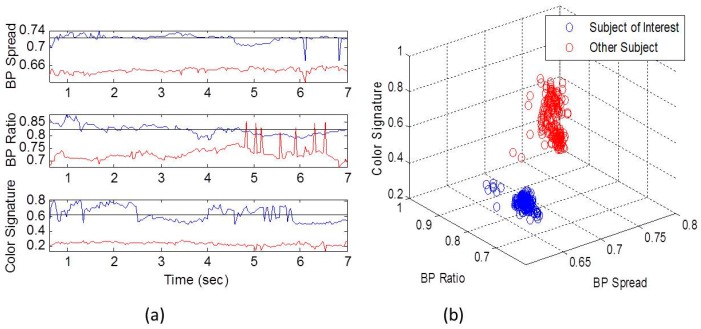
The features in time domain in calibration period (**a**), where the SoI is in blue color, and the other subject is in red color; and in the related feature space (**b**). The two subjects are well separated in both time domain and in feature domain in all the features.

The KS was established using the two static features (sum of the four limbs, and the shoulders distance divided by the spine length), and color. The color features was the ratio of Red/Blue colors of the torso, multiplied by the RGB color intensity. The static KS of the two subjects is described in Figure 4. Due to the static conditions, there were no frames lost. Figure 4a shows the four features of BPs’ spread, ratio, and color intensity, and ratio, as were derived in the calibration over a 7 s calibration window. It can be seen that the SoI (blue color) is well separated from the other subject for all features. The BP’s ratio is noisier than the BP spread (Figure 4a), since the division operation enhances noise, even in the relatively static conditions of the calibration. The correlation coefficient between the BPs’ spread and the BP’s ratio is weak, having value of 0.21. This strengthen the prior assumption regard the two dimension knowledge, and indicate that the ratio holds complementary information about the body proportions, which is not included in the BP spread.

### 3.3. Gait Tracking

**Figure 5:**
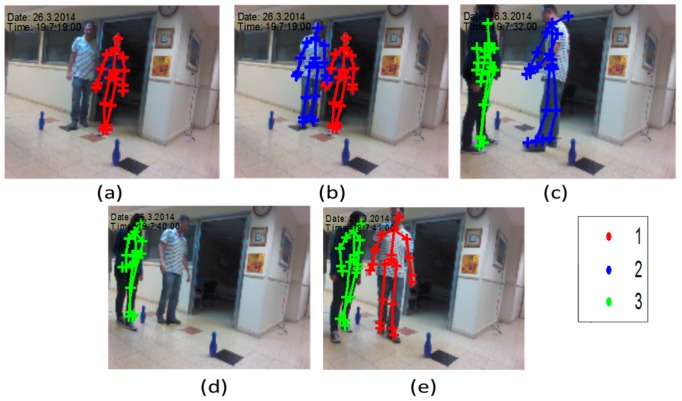
The five different subject indexes assignments, which represent different KIs, for the two subject as of the Kinect device: the other subject assign to (**a**) the first index; (**b**) the SoI assigned to index 2; (**c**) the other subject assigned to index 3; (**d**) the SoI loses its index; (**e**) and assigned again with index 1. The same subject assignments are multiplexed, due to the lack of ability of Kinect to distinguish between the different subjects in different scenes. An identification of the SoI is essential in order to analyze the SoI motion.

The Kinect tracking system software assigns to each new KI (Kinect instance of a subject) a KI serial number in the active set. Figure 5 describes the Kinect subject assignments for the two subjects (the SoI and the other subject). Five different KIs, share three different subject serial numbers in the Kinect active set at a 30 s recording session. As seen, the first assignment (first KI), is of the other subject (its skeleton is marked by red color), as shown in Figure 5a. Then, in a very short time after, the SoI is identified (second KI), and is assigned by an index number 2 (Figure 5b, blue color). After a short walk, the first subject was excluded from the active set list (due to shadowing), and when identified again (a third KI), it is assigned by index number 3 (Figure 5c, green color). A short time after, the other subject is assigned again (Figure 5d, green color). Last, the SoI is assigned by the available index number 2 (red color, fifth KI in Figure 5e). This multiplex use of indexes illustrates the need for applying an identification algorithm to identify the SoI’s KIs, to enable extracting only the SoI’s features for analysis.

**Figure 6:**
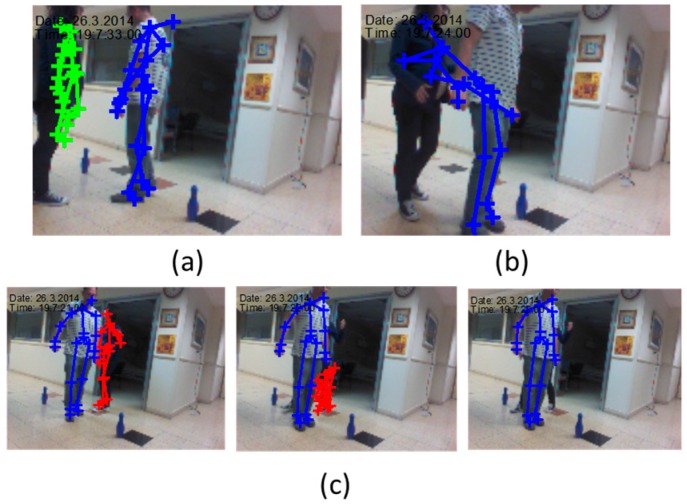
Artifact examples: (**a**) Skeleton out of proportions; (**b**) Skeleton wrong merge; (**c**) Skeleton distortion due to shadowing.

The Kinect motion tracking artifacts in real-time conditions are demonstrated in Figure 6. The artifacts are due to its range and line-of sight limitations. The three main artifacts are: (1) Skeleton distortion, and BP length change when out of Kinect effective range (Figure 6a); (2) wrong skeleton merge due to near subject (Figure 6b); (3) skeleton distortion due to shadowing (Figure 6c).

**Figure 7:**
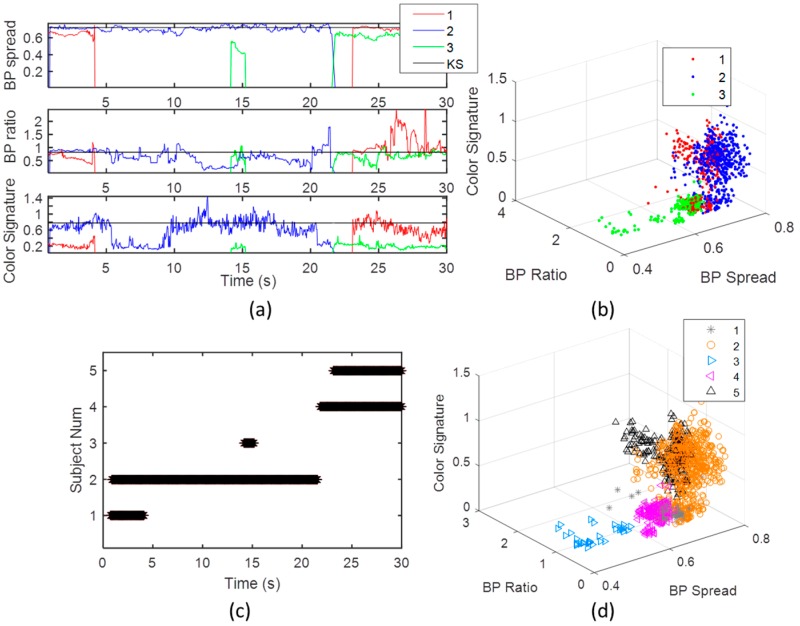
The five KIs and their corresponding three Kinect subject assignments in time (**a**); their corssponding mapping to Kinect indexes in the active set (**b**); the “life duration” of the five KIs (**c**); and the KIs in feature space (**d**).

Figure 7 shows the five KIs features of the two subjects in time and feature spaces. Figure 7a, describes the features in time domain, and Figure 7b in feature space. It is seen that the two subjects’ index assignments are multiplexed, and KI 3, and 5, include two different subjects. The KIs’ features, mainly the skeleton ratio, are distorted and hence represent a low tracking quality, or a noisy KI. Figure 7b indicates on the relatively high spread of the features. A first stage is to separate the Kinect subjects’ indexes to five separate KIs. Figure 7c,d, show the KI life duration in time, and in the feature space, respectively. The average subjects’ life duration in this real-time setup is around 10 s. The third KI (at around 15 s from the start) has as very short duration.

These five separated KIs’ features, are input to a classifier. The classification of the KIs to the SoI, other subject (not in the data base), or noise, is obtained by minimization of the criterion in Equation (8) using Euclidean Metric. Assuming that the data for different subjects has a Multi-Gaussian distribution, which can be justified by the calibration results in Figure 3b, a classifier that minimizes the error mean criterion, would be the k-mean classifier, with k equal to 2 (two subjects in the scene). In our case, different than radar or sonar system, the clusters are the KIs and are known, so the main ambiguity is reduced to find the best match of the different KIs’ clusters to the SoI’s mean value. 

**Figure 8:**
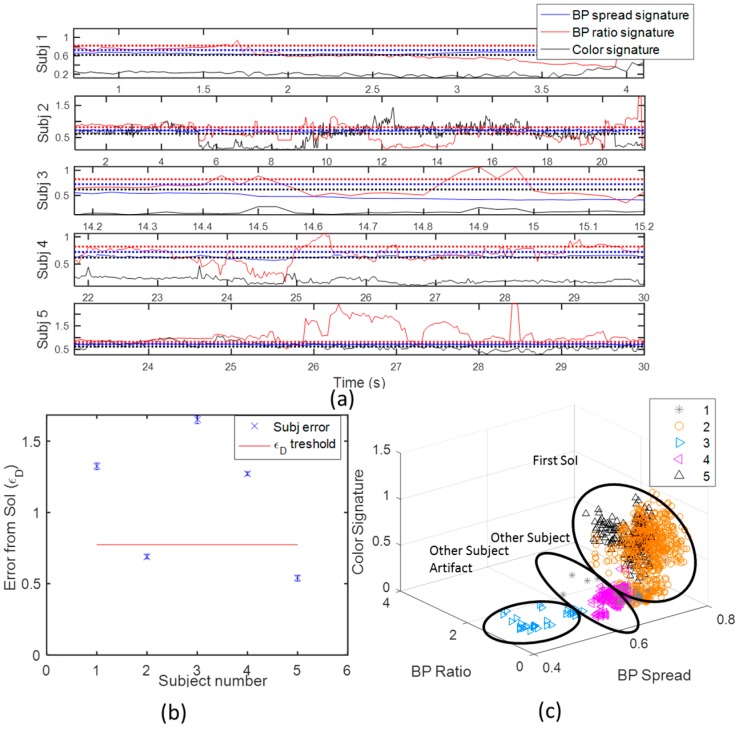
Subject classification. (**a**) Shows the different KI (Kinect Instances) compared to the SoI signatures (black line). It can be observed that objects 2, and 5, are significantly closer to the signature, and therefor seem to be related to the SoI; (**b**) Demonstrate choosing a detection threshold to maximally separate between the two subjects; (**c**) Shows the different KI mapped to the SoI, and the other subject. Note, that the other subject third instance can be seen as a noisy KI of the other subject.

Figure 8 describes this mapping process. The error of each KI in its life time is shown in Figure 8a. In all KIs, it seems that its distance from the KS is significant for most features. The equally combined Euclidean error for all features mean and standard error are shown in Figure 8b. The subject that are closer to the KS in the mean of minimal square root error (MMSE), are chosen to be the one that relate to the SoI. The KIs’ clusters after classification are shown in Figure 8c. This classification is consistent with the calibration results in Figure 3c, as expected. A *t*-test measure indicated on a very significant separation between the two subjects’ classified clusters (more than 99% right estimations). The significance between the features is also very high (*p*-value $<10^{-8}$ for all features).

**Figure 9:**
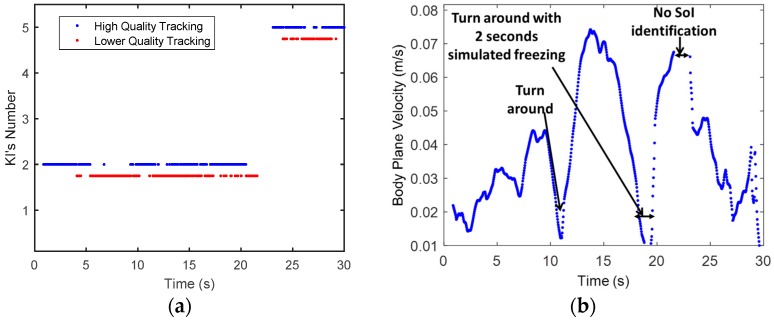
The SoI’s gait experiment results. (**a**) Shows the SoI’s Quality of tracking (KSs’ 2 and 5), and (**b**) Shows the SoI’s Body plane (x-y) velocity over time. It is seen that there are continuous burst periods (5–10, 12–13, 20–22), the skeleton is distorted from its reference KS, and hence the tracking quality is low. These bursts correspond to the artifacts shown in Figure 5 of shadowing, merge of skeletons, and going in and out of the Kinect range; (**b**) shows the SoI’s ground plane velocity. The nulls in the plane velocity, fits the tagging of turn arounds as was derived from the Kinect video color image at around 11, and 18–20 s.

The deviation of the features from their static KS can be used to obtain estimation quality. The binary two-level quality (high or low) in Equation (7) is applied on the SoI’s KIs and shown in Figure 9b. The quality of tracking is not significantly high, with 36%, and 18% low tracking quality for the first and second KIs, respectively. This ratio reflects the relatively high distortion rate of the skeleton in the experiment.

To demonstrate the use of kinematic features for kinematic analysis, the body plane velocity (*x*-*y* plane), $v_{x-y}^{m}$, was derived using Equation (8). A 10 taps (0.33 s) low pass filter (moving average with regression) was applied on the velocity to mitigate over the lower quality tracking and very rapid movements. Figure 9b shows the SoI’s plane velocity with the body plane velocity after filtering. The first turnaround in the experiment can be captured by the decreasing following by the increasing in the ground velocity (pointed by blue arrow). The second turnaround has around 3 s of zero velocity, and capture the simulated FOG. The subject *x*-*y* mean location is less sensitive to skeleton distortion compared to the joint’s coordinates or their ratio, since it is projection to *x*-*y* planes, and reflect the center of the mass of the all skeleton. Thus this dynamic feature might be used in gait analysis even at low quality tracking. These features have a potential to detect spontaneous stops, collect gait statics, and assist in detection of times suspected as FOG [19]. From the nulls at around 11, and 18–20 s, it seems that the body plane velocity feature is less sensitive to skeleton distortion, and can be used in gait analysis even at low quality tracking.

### 3.4. Tapping Activity Tracking

**Figure 10:**
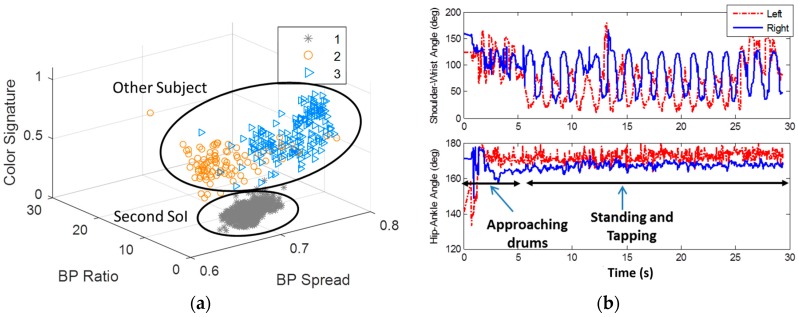
Tapping experiment results. (**a**) shows the SoI of the first KI in the tapping experiment. Like in the gait experiment it is well separated in the feature space, and its tracking quality, due to its optimal range is high; (**b**) shows upper and lower limbs orientation over time in tapping experiment. The lower limbs indicate on approaching phase with two gait cycles of length of around 4 s, and then the tapping stage begins, with periodic upper limbs activity. The subject in background did not affect the estimations, due to the optimal SoI location, and the line of sight conditions.

The capability of the Kinect to extract dynamic features that can be used to assess human activity, and its performance in home-like environment, was demonstrated in the third experiment tapping experiment (Figure 3c). Figure 10a shows the different KIs in this experiment. The longest KI in was the SoI, and the two others were of the other interfering subject in the background. The quality of the tracking is very high (more than 99% high quality skeleton estimations), due to the optimal range and the line of sight conditions. Based on the research in [33,39], the high quality Kinect estimations, can be based to form reliable dynamic features that can be used for physical activity.

The dynamic features for the lower and upper limbs’ angles as derived from the SOI’s joints using Equation (7) are shown in Figure 10b. The lower limbs angles indicate on approaching phase with two gait cycles of length of around 4 s. Then the relatively constant lower limbs angles indicate on stopping. At this point, the upper limbs start periodic movement, which indicate on the tapping activity. The subject in background did not affect the estimations, due to the optimal SoI location, and the preservation of line of sight conditions. The characteristics of the tapping speed, symmetry, and response time, can be used to evaluate PD severity [34] in an objective manner.

## 4. Conclusions

In this paper, we suggest a new procedure and computational techniques to handle some of the existing known drawbacks of the Kinect System (v1. and v2): (1) The Kinect does not include subject identification, and thus can assign the Kinect estimations to the wrong subject; (2) it can suffer from distortions due to limited coverage range and shadowing that can result in degradation in the subject kinematics assessment’s quality. 

The work provided a limited set of static skeleton based features of ratio and length of different BPs that can be used based on their time-invariance characteristics to identify subjects. Relatively a small number of these features were used to build a KS Kinect Signature of the Subjects of Interest (SoIs). Distorted estimations were detected and excluded in the feature domain based on feature prior knowledge of consistency over time, and enabled identification of the KIs of the SoI using k-mean classification algorithm. Then, kinematic features based on the SoI’s joints’ estimation were derived to demonstrate a Kinematics analysis. 

The feasibility of the suggested technology was shown by three experiment sets. In the first one, the validity of the features to identify different subjects was verified using a DFA classifier. The identification accuracy of five different subjects was around 99% with all the 34 skeleton features, and reduced to around 90% with only two skeleton features. The second experiment demonstrated the applicability of methods in a challenging environment, in which the SoI, the other subject’s KI, and a distorted skeleton, were successfully detected. It enabled merging the different SoIs’ KIs to one, and to detect and remove if needed low quality tracking features and time instances. The third experiment set demonstrated the use of dynamic features like body plane velocity and relative upper and lower limbs angles, to collect kinematic statistics for advanced kinematic analysis. Similar data analysis and implementation scheme can be tailored to the case of multiple Kinect sensors to increase coverage. The KS estimation and reliability criterion can be applied on each sensor separately, and then combined by choosing the Kinect sensor with the best subject has maximal observance. More complex approaches can be used in future to aggregate multiple Kinect sensors to single joints’ estimations. 

In future, the suggested technology should be tested with more activities and more subjects; more features, like pose, and facial features, should be added to the KS feature set to increase identification accuracy; dynamic features such as BPs’ standard deviation, changes in posture, stride length, asymmetry of gait, can be aggregated to the KS; more advanced feature selection and classification algorithm can be applied; multiple synchronized Kinect sensors to increase tracking accuracy and coverage is planned to be used; smart interpolation with additional sensor data should be used; and clinical experiments with different patients with different diseases to evaluate patients’ condition at home environment should be conducted.

Utilization of the suggested system and procedure can enable the Kinect to be a reliable resource for collecting at home kinematic information related to daily life activities. The Kinect analysis results can then be sent in real time directly from the patient home, to a patient database in the hospital for use in continuous monitoring and follow-up treatments. This technology can further assist in detection of risk-life situations like fall detection, fainting, or stroke, of elderly people at their home.

## Appendix A

**Table A1:** List of *N*_f_ = 34 features.

Feature Type		
BPs Lengths	BPs Ratios	BPs Spread
1. SpineShoulder to Head	22. (ShoulderRight to ShoulderLeft)/(SpineShoulder to SpineBase)	33. Approximate height
2. ShoulderRight to ElbowRight	23. (SpineShoulder to SpineMid)/(SpineMid to SpineBase)	34. Body limbs spread
3. ShoulderLeft to ElbowLeft	24. (Head to Neck)/(SpineShoulder to SpineBase)	
4. WristRight to ElbowRight	25. (ShoulderRight to WristRight)/(SpineShoulder to SpineBase)	
5. WristLeft to ElbowLeft	26. (HipRight to AnkleRight)/(SpineShoulder to SpineBase)	
6. HipRight to KneeRight	27. (HipRight to AnkleRight)/(ShoulderRight to WristRight)	
7. HipLeft to KneeLeft	28. (HipRight to HipLeft)/(ShoulderRight to ShoulderLeft)	
8. KneeRight to AnkleRight	29. (HipRight to HipLeft)/(HipRight to AnkleRight)	
9. KneeLeft to AnkleLeft	30. (HipRight to HipLeft)/(ShoulderRight to WristRight)	
10. WristRight to HandRight	31. (ShoulderRight to ShoulderLeft)/(HipRight to AnkleRight)	
11. WristLeft to HandLeft	32. (ShoulderRight to ShoulderLeft)/(ShoulderRight to WristRight)	
12. AnkleRight to FootRight		
13. AnkleLeft to FootLeft		
14. Head to Neck		
15. Neck to SpineShoulder		
16. SpineShoulder to SpineMid		
17. SpineMid to SpineBase		
18. SpineBase to HipRight		
19. HipRight to HipLeft		
20. ShoulderRight to ShoulderLeft		
21. SpineShoulder to SpineBase		

## Appendix B

**Table B1:** The confusion matrix using DFA classifier after artifact removal for all *N*_f_ = 34 features.

Subject Number				
**1st**	**2nd**	**3rd**	**4th**	**5th**
**100**	0	0	0	0
0	**100**	0	0	0
0	0	**99.9**	0.7	0.3
0	0	0	**98.5**	1.5
0	0		0.74	**97.9**

**Table B2:** The confusion matrix using DFA classifier after artifact removal for 10 randomly chosen features permutation using domain knowledge (5 joints’ ratio, 5 joints’ length).

Subject Number				
**1st**	**2nd**	**3rd**	**4th**	**5th**
**96.9**	0	0	0	0
0	**98.3**	0	0	0
2.99	1.65	**98.9**	3.02	0.21
0	0.06	0.79	**96.56**	1.49
0.11	0	0.28	0.41	**98.3**

**Table B3:** The Confusion matrix using DFA classifier after artifact removal for the 2 chosen features (1 joint ratio, 1 joint length).

Subject Number				
**1st**	**2nd**	**3rd**	**4th**	**5th**
**75.57**	0	0	2.94	0.5
5.6	**100**	0	0	0
0	0	**93.6**	1.58	7.1
11.94	0	3.11	**95.12**	4.1
6.89	0	3.29	0.4	**88.3**
